# Vertebral defect, anal atresia, cardiac defect, tracheoesophageal fistula/esophageal atresia, renal defect, and limb defect association with Mayer-Rokitansky-Küster-Hauser syndrome in co-occurrence: two case reports and a review of the literature

**DOI:** 10.1186/s13256-016-1127-9

**Published:** 2016-12-21

**Authors:** Thomas Bjørsum-Meyer, Morten Herlin, Niels Qvist, Michael B. Petersen

**Affiliations:** 1 Department of Surgery, Odense University Hospital, Sdr. Boulevard 29, Odense, C 5000 Denmark; 2 University of Southern Denmark, Campusvej 55, Odense, M 5230 Denmark; 3 Department of Clinical Genetics, Aalborg University Hospital, Ladegårdsgade 5, Aalborg, 9000 Denmark; 4 Department of Clinical Medicine, Aalborg University, Sdr. Skovvej 15, Aalborg, 9000 Denmark

**Keywords:** Congenital abnormalities, Syndrome association, VACTERL association, Mullerian aplasia, Rare diseases

## Abstract

**Background:**

The vertebral defect, anal atresia, cardiac defect, tracheoesophageal fistula/esophageal atresia, renal defect, and limb defect association and Mayer-Rokitansky-Küster-Hauser syndrome are rare conditions. We aimed to present two cases with the vertebral defect, anal atresia, cardiac defect, tracheoesophageal fistula/esophageal atresia, renal defect, and limb defect association and Mayer-Rokitansky-Küster-Hauser co-occurrence from our local surgical center and through a systematic literature search detect published cases. Furthermore, we aimed to collect existing knowledge in the embryopathogenesis and genetics in order to discuss a possible link between the vertebral defect, anal atresia, cardiac defect, tracheoesophageal fistula/esophageal atresia, renal defect, and limb defect association and Mayer-Rokitansky-Küster-Hauser syndrome.

**Case presentation:**

Our first case was a white girl delivered by caesarean section at 37 weeks of gestation; our second case was a white girl born at a gestational age of 40 weeks. A co-occurrence of vertebral defect, anal atresia, cardiac defect, tracheoesophageal fistula/esophageal atresia, renal defect, and limb defect association and Mayer-Rokitansky-Küster-Hauser syndrome was diagnosed in both cases.

We performed a systematic literature search in PubMed ((VACTERL) OR (VATER)) AND ((MRKH) OR (Mayer-Rokitansky-Küster-Hauser) OR (mullerian agenesis) OR (mullerian aplasia) OR (MURCS)) without limitations. A similar search was performed in Embase and the Cochrane library. We added two cases from our local center.

All cases (*n* = 9) presented with anal atresia and renal defect. Vertebral defects were present in eight patients. Rectovestibular fistula was confirmed in seven patients. Along with the uterovaginal agenesis, fallopian tube aplasia appeared in five of nine cases and in two cases ovarian involvement also existed.

**Conclusions:**

The co-occurrence of the vertebral defect, anal atresia, cardiac defect, tracheoesophageal fistula/esophageal atresia, renal defect, and limb defect association and Mayer-Rokitansky-Küster-Hauser syndrome is extremely rare. This group of patients has unusual phenotypic characteristics. The long-term outcome after treatment of defects is not well reported. A single unifying cause is not known and the etiology probably includes both genetic and non-genetic causes. We stress the importance of future studies to optimized treatment, follow-up, and etiology.

## Background

The vertebral defect, anal atresia, cardiac defect, tracheoesophageal fistula/esophageal atresia, renal defect, and limb defect (VACTERL) association and Mayer-Rokitansky-Küster-Hauser (MRKH) syndrome are rare conditions. The co-occurrence of the VACTERL association and MRKH syndrome is extremely rare and has only been casuistically reported. Even with optimal surgical corrections of malformations, patients affected by the VACTERL association can face medical challenges throughout life such as back pain (scoliosis), fecal incontinence (anal atresia, AA), and functional impairment (limb anomalies) [1]. The medical challenges are highly dependent on type and severity of the specific malformation. Women affected by the MRKH syndrome are unable to bear children and the syndrome may have a long-lasting negative impact on their level of psychological distress and self-esteem [2]. Reporting cases of rare conditions is important to expand knowledge on possible etiological factors, treatment, and outcome. We have treated two patients at our center with the VACTERL association and MRKH co-occurrence, which add further knowledge to the condition. To the best of our knowledge a possible embryologic or genetic cause in the development of the VACTERL association and MRKH syndrome has not been addressed in the literature so far. We aimed to collect existing knowledge in embryopathogenesis and genetics in order to discuss a possible link between two rare disease entities.

Our objectives were:

- To present two cases with the co-occurrence of the VACTERL association and the MRKH syndrome from our surgical center.
- To perform a systematic review of the literature to detect cases with the co-occurrence of the VACTERL association and the MRKH syndrome in order to characterize patients regarding clinical presentation, outcome, and treatment.
- To discuss possible embryopathogenesis and genetics in patients with the co-occurrence of the VACTERL association and the MRKH syndrome.

### VACTERL association

VACTERL association (Online Mendelian Inheritance in Man, OMIM, 192350) is the co-occurrence of birth defects including vertebral defect, AA, cardiac defect, tracheoesophageal fistula (TEF)/esophageal atresia, radial and renal dysplasia, and limb defects. It is generally accepted that the diagnostic criteria are the occurrence of at least three of the mentioned birth defects. The prevalence has been estimated to be 1:10,000 to 1:40,000 in live births [3–5]. Most cases of VACTERL appear sporadic although familial occurrence has been described. Genetic factors have been proposed but so far no single gene mutation or chromosomal anomaly has been identified as the unifying cause [6]. Vertebral anomalies have been reported in 60 to 80% of patients [7–12] and are often accompanied by rib anomalies. Typical vertebral anomalies are hemivertebrae, vertebral fusions, and supernumerary or absent vertebrae. There was a great variety in the reports of the severity of vertebral malformations [9, 12]. A common clinical presentation is scoliosis [13]. AA is reported in 55 to 90% of patients with VACTERL association [7–11]. In contrast to vertebral anomalies, AA is often recognized during the immediate clinical examination after birth. In more subtle cases AA may present later as constipation, which appears in up to 21% of the patients [14]. Some clinicians require an imperforate anus to address it as a VACTERL association while others allow minor AA to be included. Cardiac malformations are also common and observed in 40 to 80% of patients [9–11]. They range from life-threatening malformations, which require complicated surgical procedures, to small asymptomatic defects which are discovered incidentally [10, 11, 13]. A persistent ductus arteriosus and foramen ovale should not be registered as a defect in the VACTERL association [1]. TEF is reported in 50 to 80% of patients with VACTERL [7–11]. TEF is a potentially life-threating condition and often requires surgery in the first few days of life. The prevalence of renal malformations is 50 to 80%, of which there is a great variation in severity [8–11]. They present as horseshoe kidney, cystic kidney, dysplastic kidney, or unilateral or bilateral agenesis. Limb anomalies are traditionally defined as radial anomalies including aplasia/hypoplasia of the thumb. Other limb anomalies have also been described, for example anomalies of lower extremities. They are reported in 40 to 50% of patients with VACTERL [3, 7, 8, 10, 12, 15]. The first sign of the VACTERL association may be the presence of a single umbilical artery diagnosed prenatally and this finding should lead to a comprehensive examination for other defects in the VACTERL association [16, 17].

### MRKH syndrome

MRKH syndrome (OMIM 277000) is a congenital disorder of the Müllerian ducts, characterized by agenesis or aplasia of the uterus and upper two-thirds of the vagina in females with normal secondary sex characteristics (thelarche and pubarche) and a normal karyotype (46,XX) [18]. Prevalence has been estimated to be 1 in 5000 live female births [19, 20] and MRKH syndrome is considered the second most common cause of primary amenorrhea after ovarian failure [21]. Patients typically present in their adolescence with complaints of primary amenorrhea; however, other complaints include lower abdominal pain, dyspareunia, and infertility. MRKH syndrome is classified into three groups [22]: type I/typical MRKH is characterized by isolated uterovaginal agenesis, type II/atypical MRKH is associated with renal and ovarian malformations, and the third type is Müllerian duct aplasia, renal aplasia, and cervicothoracic somite dysplasia (MURCS; OMIM 601076) association with renal, skeletal, and cardiac malformations, and hearing impairment. However, the definition and application of this classification in the literature are still inconsistent. The cause(s) of MRKH syndrome still remains unknown. Reports of discordance in monozygotic twins have suggested a non-genetic etiology. Previously, diethylstilbestrol (DES) and thalidomide were suspected as possible teratogenic causes [23, 24]; however, increasing reports of familial occurrence of MRKH syndrome and its associated extragenital malformations [25] have led to several investigations searching for a genetic etiology. Most studies conducted so far have found no clear association. Mutations in the *WNT4* gene have been associated with uterovaginal agenesis in virilized females [26] – an entity believed to be different from the MRKH syndrome. The management of MRKH syndrome involves counselling about the functional and psychosexual impacts and the creation of a neovagina in order to allow sexual intercourse. The American College of Obstetricians and Gynecologists (ACOG) recommends vaginal dilatation as first choice therapy due its few complications, but several other surgical procedures are also being used [27].

## Case presentation

### Patient medical report – case 1

**Fig. 1:**
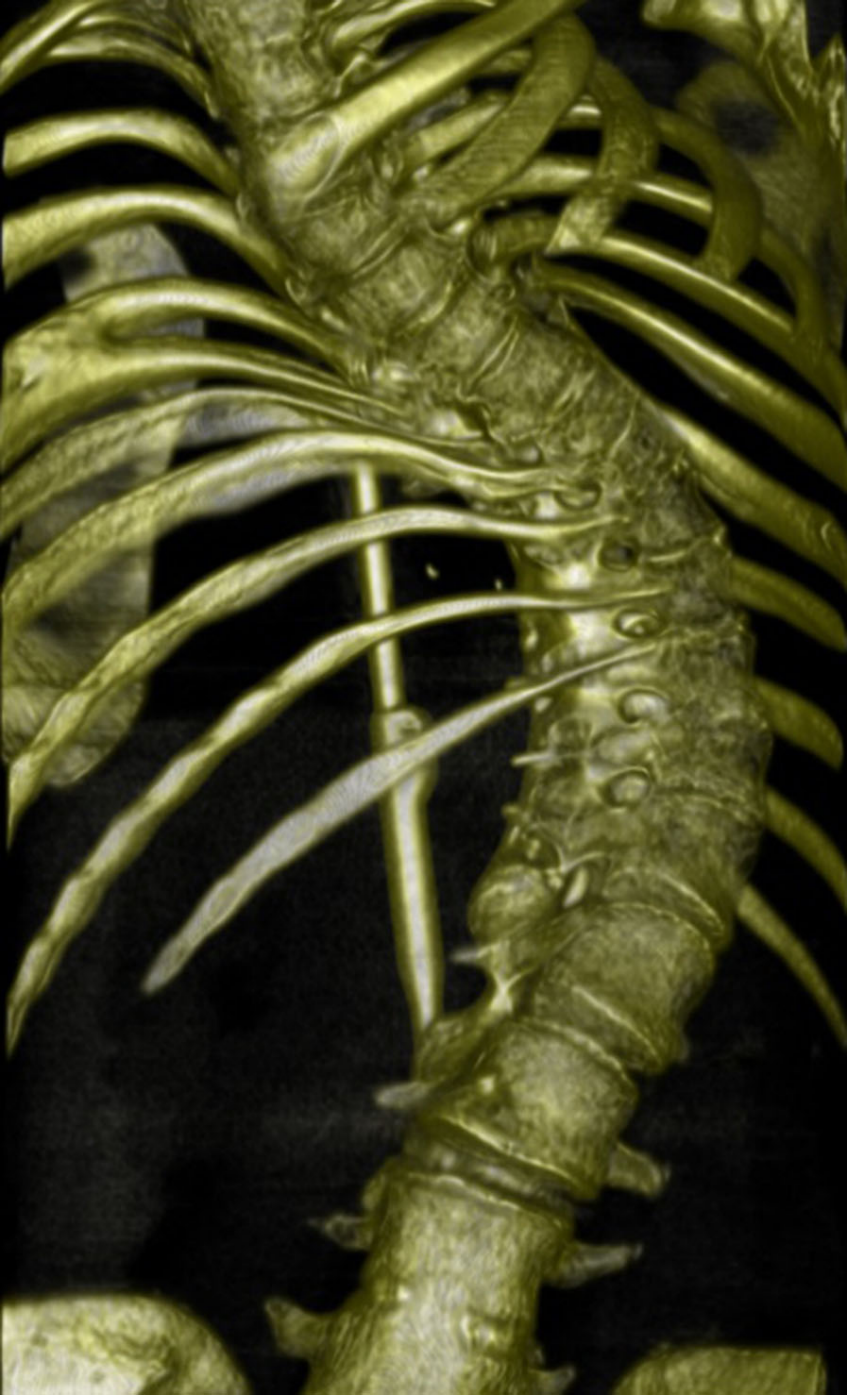
Case 1, computed tomography reconstruction of the spine showing a thoracolumbar scoliosis

A white girl was delivered by caesarean section at 37 weeks of gestation due to suspected mechanical disproportions. Her family history was without known inborn abnormalities and consanguinity. Her birthweight was 2600 g and her Apgar scores were 10/1 and 10/5. A physical examination revealed AA. An analysis of the prenatal amniotic fluid showed a normal female karyotype. Prenatal ultrasound (US) showed left-sided renal cysts and a TEF was suspected. Plain X-ray indicated esophageal atresia with TEF and high AA. Meconium was observed from her vaginal introitus. A colostomy was opened during her first day of life together with an end-to-end anastomosis of her esophagus and closure of the TEF. Ultrasonography during her fifth day of life showed a left hydronephrosis and three communicating renal cysts with a normal right kidney. A renography performed at 2 years of age showed no function of her left kidney. A late posterior sagittal anorectoplasty (PSARP) was performed when she was 3-years old. Perioperatively a vaginal aplasia was found and no uterus was found. Two months after the PSARP the colostomy was closed and a left nephrectomy performed. She developed a thoracolumbar scoliosis confirmed by a computed tomography (CT) scan (Fig. 1) which was corrected during childhood and adolescence through several operations. At the age of 15 years vaginal dilations treatment was started. At 20 years of age she was admitted to hospital with lower abdominal pain. A transvaginal US showed an abscess at her vaginal top, which was treated with transvaginal drainage and antibiotics. The following 4 weeks were uneventful and it was decided to attempt a surgical extension of her vagina. The procedure was complicated by a lesion of her bladder neck which was treated conservatively with a urethral catheter for 4 weeks without further complications. A magnetic resonance (MR) scan afterwards showed no restoration of the abscess. Her left ovary was absent and her fallopian tube appeared rudimentary on the left side. A normal ovary was documented on her right side. In 2015 at the age of 22 years a new attempt at dilations was made, which was now preceded by a small incision at the top of her vaginal pouch. The dilation treatment resulted in an 8 cm-long vagina.

She fulfilled the criteria for VACTERL association with vertebral anomalies, AA, TEF, and kidney defect. Her diagnostic criteria for MRKH syndrome were aplasia of the vagina, absent uterus, and a multicystic dysplastic left kidney.

### Patient medical report – case 2

**Fig. 2:**
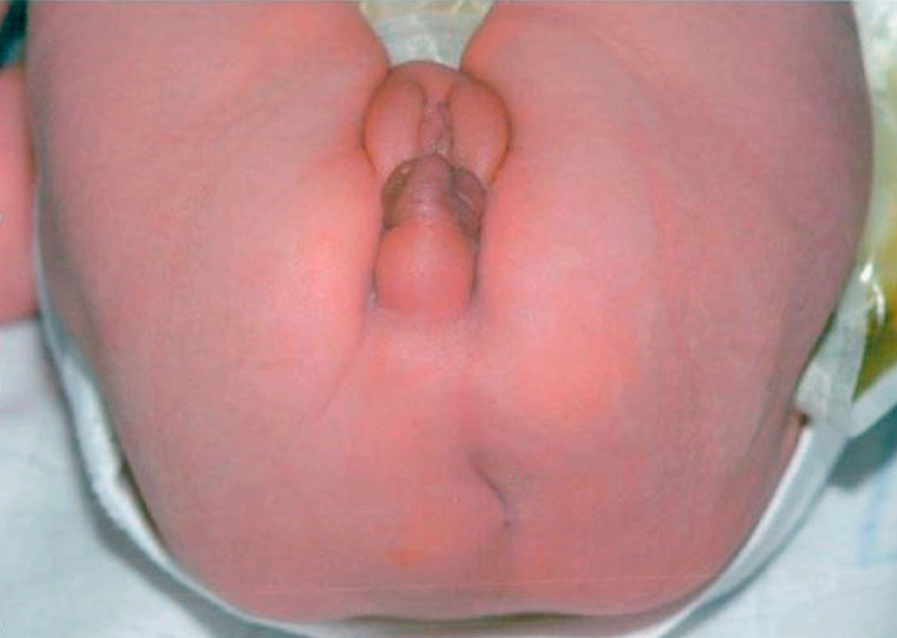
Case 2 (6-days old), clinical photograph of the patient in the supine position showing an absent anus. Fistula opening is not presented

A white girl born at a gestational age of 40 weeks with a birthweight of 3088 g presented with AA (Fig. 2) and a fistula opening near her vaginal introitus. Her family history was without known inborn abnormalities and consanguinity. Her Apgar scores were 8, 9, and 10 after 1, 5, and 10 minutes respectively. The pregnancy was uneventful. A diverging colostomy was created at 11 days of age. Perioperatively a remnant of vagina was found and the uterus was absent. Normal fallopian tubes and ovaries were demonstrated on both sides. A duplex kidney was found on her right side with hydroureter. A severe sacral dysgenesis was suspected clinically. She had a normal karyotype. PSARP was performed at the age of 4 months. After 6 months the colostomy was closed. A MR scan at 21 months of age revealed a tethered spinal cord and an intradural lipoma from L4 to S2.

Despite dietary restrictions and laxatives our patient had chronic constipation and an appendicostomy for antegrade colonic enema was created at 7 years of age. She continued to have abdominal pain, nausea, and vomiting. A sigmoidoscopy showed a siphon-like configuration at her colonic anastomosis; therefore, it was decided to perform a resection of her anastomotic colon, which was complicated by anastomotic leakage necessitating an intermittent diverting colostomy.

Her criteria for the VACTERL association were tethered cord and sacral scoliosis, AA, and duplex kidney. Absent uterus and proximal vaginal agenesis were diagnostic for MRKH syndrome.

## Discussion

### Search strategy and data collection

**Fig. 3:**
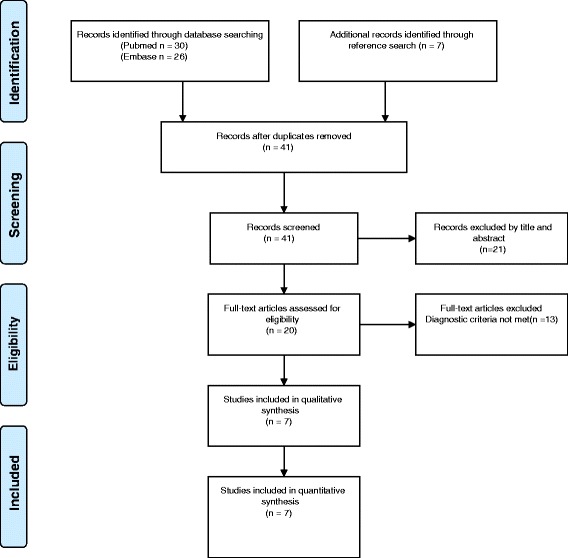
Flow diagram of record selection

A literature search was conducted in the PubMed database using the following search terms: ((VACTERL) OR (VATER)) AND ((MRKH) OR (Mayer-Rokitansky-Küster-Hauser) OR (mullerian agenesis) OR (mullerian aplasia) OR (MURCS)) from inception to 18 September 2015 (Fig. 3) without limitations. A similar literature search was performed in Embase and the Cochrane Library. Furthermore a reference search was made. Identified cases are shown in Table 1. Three authors were contacted regarding further information on their cases and one responded. To be included, the cases had to meet the diagnostic criteria for both the VACTERL association and MRKH syndrome and not fulfill the criteria for other syndromes or associations. Karyotype had to be normal but we also included cases without information on karyotype.

**Table 1:** Co-occurrence of vertebral defect, anal atresia, cardiac defect, tracheoesophageal fistula/esophageal atresia, renal defect, and limb defect association and Mayer-Rokitansky-Küster-Hauser syndrome in the literature and two cases from our local center

Authors	VACTERL features						Uterovaginal malformation	Other anomalies	Karyotype	Surgical and neovaginal procedures	Bowel and/or urogenital symptoms at follow-up
	V	A	C	T E	R	L					
Günsąr *et al.* [75]	X	X			X		UVag agenesis	Rib anomalies	46,XX	PSARP + sigmoid loop vaginoplasty (one-stage operation)	5-years old (none)
King *et al.* [76]	X	X		X	X		UVagF agenesis	Rib anomaly	46,XX	NS	Died at 3 months of age
Komura *et al.* [77]	X	X	X	X	X	X	UVag agenesis	Left facial nerve palsy, left hearing impairment, seizures	NS	PSARP	3 years (tracheostomy tube, mosapride for GERD, carbamazepine for seizures)
Linke *et al.* [78]		X	X	X	X		UVagF agenesis	Right esophageal lung, duodenal atresia	46,XX	None	Died at 4 weeks of age
Rall *et al.* [30]	X	X			X	X	UVagO agenesis	Hearing impairment, right ear tag	46,XX	PSARP, modified Vecchietti technique	3 months after modified Vecchietti operation (no vaginal symptoms)
Teo *et al.* [79]	X	X	X	X	X		UVagF agenesis	Bilateral vesico-ureteric reflux, overactive bladder	46,XX	PSARP	NS
Wester *et al.* [80]	X	X			X	X	UVagF agenesis	None	NS	ASARP + sigmoid loop vaginoplasty (one-stage operation)	18 months (laxatives)
Bjørsum-Meyer *et al.* (this report)	X	X	X	X	X		UVagFO agenesis	None	46,XX	PSARP, vaginal dilatations	22 years (vaginal dilatations)
	X	X			X		UVag agenesis	None	46,XX	PSARP	10 years (laxatives)

*A* anal atresia, *ASARP* anterior sagittal anorectoplasty, *C* cardiac anomaly, *F* fallopian tube, *GERD* gastroesophageal reflux disease, *L* limb anomaly, *NS* not stated, *O* unilateral ovary, *PSARP* posterior sagittal anorectoplasty, *R* renal and/or radial anomalies, *TE* tracheoesophageal fistula and/or esophageal atresia, *U* uterus, *V* vertebral anomaly, *Vag* vagina

Diagnostic criteria for the VACTERL association: ≥3 of the following: vertebral defect, AA, cardiac defect, TEF/esophageal atresia, renal defect, and limb defect.

Diagnostic criteria for MRKH syndrome: agenesis of the upper vagina and uterus.

### Quality assessment of case reports

The transparency and accuracy of included case reports were assessed using the CARE checklist [28]. Each of the 14 items included in the CARE checklist was denoted either 0 = missing, 1 = fair, 2 = good or not applicable. A total score of <50% of a possible maximum was denoted poor, 50 to 74% = fair and ≥75% = good. Two of the included cases were not described in case reports and the CARE checklist was therefore not applicable. Of the five quality assessed case reports, four were estimated as fair whereas one case report was estimated as poor.

### Demographics and clinical presentation

Six of the seven case reports were published in the twenty-first century whereas one study was published in 1977. Most studies (*n* = 5) were European, one was from Asia, and one was from USA.

*A single umbilical artery was not described in any of the nine cases*.

In all cases (*n* = 9) reported with the VACTERL association and MRKH syndrome anorectal malformations and renal defects were present (Table 1). In all but one case, a vertebral defect was detected. In one case all features of the VACTERL association were present. Numbers of involved VACTERL components varied from three to all six components. In seven patients a rectovestibular fistula was present.

Along with the uterovaginal agenesis, fallopian tube aplasia appeared in five of nine cases and in two cases there was also ovarian involvement. Associated anomalies were described in six out of nine cases.

### Surgical approach

PSARP was the preferred surgical technique for repair of anorectal malformations; it was applied in six out of nine patients. In one patient an anterior sagittal anorectoplasty (ASARP) was preferred. The surgical techniques for uterovaginal malformations were sigmoid loop vaginoplasty (*N* = 2), modified Vecchietti, and vaginal dilations. Two patients died within the first 3 months of life and no surgical repair performed.

## Conclusions

### Clinical presentation and treatment strategies

All included cases with the VACTERL association and MRKH syndrome presented with AA. Totonelli *et al.* assessed the outcome in patients with AA as part of the VACTERL association (VACTERL+) [29]. In 174 patients with AA, 31 (18%) patients fulfilled criteria for the VACTERL association. Rectovestibular fistula was present in seven (23%) of these patients (VACTERL+) which was similar to patients without VACTERL. The poorer functional outcome in patients with VACTERL+ might be related to a higher incidence of spinal anomalies. In all cases reported with coexisting VACTERL and MRKH, AA was present and eight out of nine cases also had a vertebral defect.

In a cohort of 346 patients with MRKH syndrome, Rall *et al.* only found the presence of AA in three patients whereas one patient presented with the VACTERL association [30]. The high incidence of AA in patients who presented with the co-occurrence of the VACTERL association and MRKH syndrome is in accordance with the high incidence of AA in patients with VACTERL. The recommended surgical technique for repair of anorectal malformations with rectovestibular fistula is a PSARP, which is reported to have an excellent functional outcome in highly specialized centers and no associated defects [31]. According to our case report, PSARP was the preferred surgical approach for the repair of anorectal malformation in six of the nine cases presented.

In our detected cases with the co-occurrence of the VACTERL association and MRKH syndrome sparse information exist regarding treatment of the vaginal aplasia. This can be attributed to a lack of follow-up in reported cases. Non-surgical creation of a neovagina is the preferred treatment option according to ACOG [32]. Treatment of MRKH with vaginal dilations has been reported with anatomical and functional success in 90 to 95% of patients [33, 34]. The first-line approach with vaginal dilations as well as surgical treatment is best planned when the patient is emotionally mature during late adolescence or young adulthood. Surgical treatment is an option in patients who are unsuccessfully treated with vaginal dilations. Ongoing postoperative vaginal dilations or vaginal intercourse can maintain adequate vaginal dimensioning. Several surgical procedures exist to create a neovagina, such as the Abbe-McIndoe operation, the Vecchietti procedure, and modifications of these procedures. No consensus exists on the preferred surgical technique to achieve the best functional outcome [35].

### Embryopathogenesis and genetics of VACTERL association and MRKH syndrome

Cases of VACTERL association and MRKH syndrome co-occurrence are extremely rare, and to-date no unifying cause has been reported in these cases. It is therefore imperative to consider current knowledge of each entity when discussing possible etiologies.

The organ systems affected in VACTERL association develop at different stages of organogenesis. The vertebrae develop early (23 to 32 days) in contrast to the formation of anorectal structures, which occur late in organogenesis (45 to 56 days). With this in mind several potential explanations need to be considered, as suggested by Stevenson and Hunter [36]: (1) teratogenic exposure throughout the entire organogenesis, (2) an inaugural malformation disturbing the development of other organs (malformation sequence), (3) disturbance of molecular pathways or single gene mutations involved in the formation of multiple organ systems, and (4) a general disturbance of the developmental process (for example vascular insufficiency). Most cases of VACTERL association are sporadic (~90%) [37] and high discordance rates in both monozygotic and dizygotic twin pairs have been reported [38] suggesting the impact of germline genetics to be minor in the majority of cases. Reports of true familial cases of VACTERL association (≥3 component features; CFs) are rare; however, the prevalence of single CFs in relatives of patients with VACTERL association is ~10% suggesting a genetic etiology to some extent [37, 39]. The deletion of genes in the Sonic hedgehog pathway (for example *SHH*, *GLI*, and *HOXD13*) modeled in mice mimics many of the same malformations as found in VACTERL association, which have resulted in a profound interest in these genes [40]. However, only mutations of *HOXD13* [41] and *FOXF1* [42, 43] have been reported in humans with VACTERL association, while *SHH* and *GLI2* mutations are associated with holoprosencephaly [40]. Furthermore, mutations in *ZIC3* have been reported in several cases of VACTERL association [43–45]. Several copy number variations (CNVs) have been reported (thoroughly reviewed by Brosens *et al.* [46]), of which recurrent *de novo* CNVs have been reported at 8q24.3 (*GLI4*) and 17q23 (*TBX2*/*TBX4*). Finally, a few cases of VACTERL association and mitochondrial dysfunction have been reported [47]. To-date, the only well-established environmental cause of VACTERL association in humans is maternal diabetes mellitus [1]. However, rodent models exposed to adriamycin express many VACTERL-like features supporting the influence of teratogenic exposures [48]. In contrast to VACTERL association, the etiology of VACTERL-hydrocephalus is well established, it is associated with Fanconi anemia (both recessive and X-linked mutations) [49].

As in the case of VACTERL association, the etiology of MRKH syndrome/MURCS association remains elusive. Both *in utero* exposure to teratogens, epigenetics, and genetics have been considered as possible explanations. Decades ago, MRKH syndrome was thought to be caused by teratogens such as thalidomide [24]. The idea of a non-genetic etiology is supported by several reports of discordance in monozygotic twins [50–53] and recently by a report of a phthalate-induced rodent model phenocopying MRKH syndrome [54]. However, most investigations conducted in the last decade have focused on the search of genetic causes due to increasing reports of familial clustering suggesting a low-penetrant autosomal dominant or polygenic inheritance [55]. Various promising CNVs have been reported located at 1q21.1 (*RBM8A*) [56–58], 16p11.2 (*TBX6*) [59, 60], 17q12 (*LHX1*, *HNF1B*) [56–62], as well as 22q11 [56, 57, 63, 64] associated with DiGeorge syndrome. Although intensively studied, the outcomes of candidate gene analyses have generally been sparse [65]. Positive findings from molecular genetic analyses include mutations in *LHX1* [41, 66], *TBX6* [60, 67], *RBM8A* [67], and *WNT9B* [68, 69]. Finally, mutations in *WNT4* have been reported in females with Müllerian aplasia and hyperandrogenism [26, 70, 71], although it is thought to be an entity that differs from MRKH syndrome. However, these genetic findings only apply to a small number of patients and a more general understanding of the etiology of MRKH syndrome is still needed.

Other syndromes, with known genetic etiology, occasionally sharing features with both VACTERL association and MRKH syndrome should also be considered in the assessment of these patients. These syndromes mainly include Townes–Brocks (*SALL1*) [72], CHARGE (*CHD7*) [73], Fraser (*FRAS1*) [74], and DiGeorge syndromes (del22q11) [63, 64].

As mentioned above, several possible etiologies (both non-genetic and genetic) have to be considered in cases of VACTERL and MRKH co-occurrence. Furthermore, the close interspatial relationship between the hindgut, urogenital sinus, Müllerian ducts, and developing kidneys could suggest an inaugural malformation and subsequently disturbed development of other organs (malformation sequence). With the current knowledge of genetics in VACTERL association and MRKH syndrome only being applicable for a few patients, there is currently no clinical genetic testing available for the patients with co-occurrence. However, in cases of familial occurrence it is relevant to provide genetic counseling nonetheless. The future elucidation of cause(s) is essential to ensure a correct interpretation of these multiple organ syndromes/associations and ultimately to assist a more precise genetic counseling and adequate treatment of these patients.

### Conclusion

The co-occurrence of the VACTERL association and MRKH syndrome is extremely rare and we have only detected seven reported cases but added two from our local surgical center. This group of patients has unusual phenotypic characteristics. The long-term outcome after treatment of defects is not well reported. A single unifying cause is not known and the etiology probably includes both genetic and non-genetic causes. We stress the importance of future studies to optimized treatment, follow-up, and etiology.

## Abbreviations

AA: Anal atresia
ACOG: American College of Obstetricians and Gynecologists
ASARP: Anterior sagittal anorectoplasty
CF: Component feature
CNV: Copy number variation
CT: Computed tomography
DES: Diethylstilbestrol
MR: Magnetic resonance
MRKH: Mayer-Rokitansky-Küster-Hauser
MURCS: Müllerian duct aplasia, renal aplasia, and cervicothoracic somite dysplasia
OMIM: Online Mendelian Inheritance in Man
PSARP: Posterior sagittal anorectoplasty
TEF: Tracheoesophageal fistula
US: Ultrasound
VACTERL: Vertebral defect, anal atresia, cardiac defect, tracheoesophageal fistula/esophageal atresia, renal defect, and limb defect
